# Effectiveness of plant-based repellents against different *Anopheles* species: a systematic review

**DOI:** 10.1186/s12936-019-3064-8

**Published:** 2019-12-21

**Authors:** Amin Asadollahi, Mehdi Khoobdel, Alireza Zahraei-Ramazani, Sahar Azarmi, Sayed Hussain Mosawi

**Affiliations:** 1 Department of Medical Entomology and Vector Control, School of Public Health, Tehran University of Medical Sciences, Tehran, Iran; 2 Health Research Centre, Lifestyle Institute, Baqiyatallah University of Medical Sciences, Tehran, Iran; 3 Medical Sciences Research Centre, Ghalib University, Kabul, Afghanistan

**Keywords:** Plant, Herb, Repellent, Repellency, Systematic review, *Anopheles*

## Abstract

Plant-based repellents have been applied for generations in traditional practice as a personal protection approach against different species of *Anopheles*. Knowledge of traditional repellent plants is a significant resource for the development of new natural products as an alternative to chemical repellents. Many studies have reported evidence of repellant activities of plant extracts or essential oils against malaria vectors worldwide. This systematic review aimed to assess the effectiveness of plant-based repellents against *Anopheles* mosquitoes. All eligible studies on the repellency effects of plants against *Anopheles* mosquitoes published up to July 2018 were systematically searched through PubMed/Medline, Scopus and Google scholar databases. Outcomes measures were percentage repellency and protection time. A total of 62 trials met the inclusion criteria. The highest repellency effect was identified from *Ligusticum sinense* extract, followed by citronella, pine, *Dalbergia sissoo*, peppermint and *Rhizophora mucronata* oils with complete protection time ranging from 9.1 to 11.5 h. Furthermore, essential oils from plants such as lavender, camphor, catnip, geranium, jasmine, broad-leaved eucalyptus, lemongrass, lemon-scented eucalyptus, amyris, narrow-leaved eucalyptus, carotin, cedarwood, chamomile, cinnamon oil, juniper, cajeput, soya bean, rosemary, niaouli, olive, tagetes, violet, sandalwood, litsea, galbanum, and *Curcuma longa* also showed good repellency with 8 h complete repellency against different species of *Anopheles*. Essential oils and extracts of some plants could be formulated for the development of eco-friendly repellents against *Anopheles* species. Plant oils may serve as suitable alternatives to synthetic repellents in the future as they are relatively safe, inexpensive, and are readily available in many parts of the world.

## Background

Mosquito-transmitted diseases remain a main source of illness and death [1]. Despite decades of malaria control efforts, malaria continues to be a major worldwide public health issue with 3.3 billion persons at risk in 106 countries and territories in the tropical and subtropical areas [2]. It is one of the significant reasons for maternal and childhood morbidity and mortality, including low birth weight, stillbirths, and early infant death in sub-Saharan Africa [3]. Among 500 species of *Anopheles* mosquitoes known globally, more than 50 species can transmit malaria from the bite of the infected female *Anopheles* spp. [4]. Presently, there is no effective prophylactic anti-malarial vaccine and no suitable preventive measure other than vector control is available [5]. Thus, protection from mosquito bites is one of the best approaches to reduce the disease incidence.

The use of repellents to protect people from bites of mosquitoes previously has been acknowledged as part of an overall integrated insect-borne disease control programme [6]. Most commercial repellents are produced by using chemical components such as N, N-diethyl-meta-toluamide (DEET), Allethrin, N, N-diethyl mendelic acid amide, and Dimethyl phthalate [1]. It has been identified that chemical repellents are not safe for public health and should be used with caution because of their detrimental impacts on synthetic fabric and plastic as well as toxic reactions, such as allergy, dermatitis, and cardiovascular and neurological side effects, which have been reported generally after misapplication [4]. The frequent use of synthetic repellents with chemical origin for mosquito control has disturbed natural ecosystems and resulted in the development of resistance to insecticides, resurgence in mosquito populations, and adverse impact on non-target organisms [4, 7]. Accordingly, the idea of using natural mosquito repellent products as an alternative to develop new eco-friendly repellents could be an amicable solution to scale back the undesirable effects on environment and human health.

In recent years, interest in plant-based repellents has been revived, as they contain a rich source of bioactive phytochemicals that are safe and biodegradable into non-toxic by-products, which could be screened for insecticidal activities and mosquito repellent. Many studies have reported evidence of repellant activities of plant extracts or essential oils against malaria vectors around the world. The present systematic review was performed to reveal which plant-based repellent can be relied on to provide a prolonged and predictable protection from species of *Anopheles* mosquitoes without causing side effects on human health.

For this systematic review, all eligible studies on the repellency effects of plant-based repellants against *Anopheles* spp. published up to July 2018 were systematically searched through electronic databases PubMed, MEDLINE, Web of Science, Literature retrieval System of the Armed Forces Pest Management Board, Scopus and Google Scholar using the following Medical Subject Headings (Mesh) and keywords: (((Plant [Title/Abstract]) OR Plants [Title/Abstract]) OR herbal [Title/Abstract]) AND (botanical [Title/Abstract]) AND ((extract [Title/Abstract]) OR extracts [Title/Abstract]) AND ((“essential oil” [Title/Abstract]) OR “essential oils” [Title/Abstract]) AND (((((“Insect repellent” [Mesh]) OR repellents) OR repellent) OR repellence) OR repellency) AND ((“*Anopheles*” [Mesh]) OR “*Anopheles*” [Title/Abstract]). The search was limited to English publications. In addition, a manual search was conducted to identify further pertinent articles using references from retrieved studies.

### Eligibility criteria

Studies were included in the present systematic review if they met these criteria: (i) full-text publication was written in English, (ii) inspected the repellency effects of plant extracts and essential oils against malaria vectors, *Anopheles* spp. mosquitoes, and, (iii) reported the percentage of repellency or complete protection time. Following studies were excluded: studies exploring the repellency effect of chemical-based products, studies examining the repellency effect of animal extracts, animal studies (studies not on human subjects), articles without full texts, reviews, duplicate articles, abstracts, republished data, comments, conference papers, editorials, and studies with insufficient data. In addition, studies were excluded if the information could not be extracted. A screening of titles and abstracts followed by a full-text review was performed by two investigators. All titles and abstracts were screened by two independent investigators for eligibility. If a consensus was reached, a study was excluded or selected to full-text screening. If a consensus was not reached, another reviewer was consulted to resolve any feasible discrepancies.

### Data extraction

After identifying the eligible studies, the following data were collected from each study by application of standardized data collection form to improve accuracy and critical appraisal: the first author name, country of origin, journal details, publication year, condition of study (field or laboratory), plant name, *Anopheles* species, concentration or dose of repellents, repellency percentage and complete protection time. All data were independently extracted by two reviewers and disagreements were solved by discussion, and if necessary, a third author was involved.

**Fig. 1:**
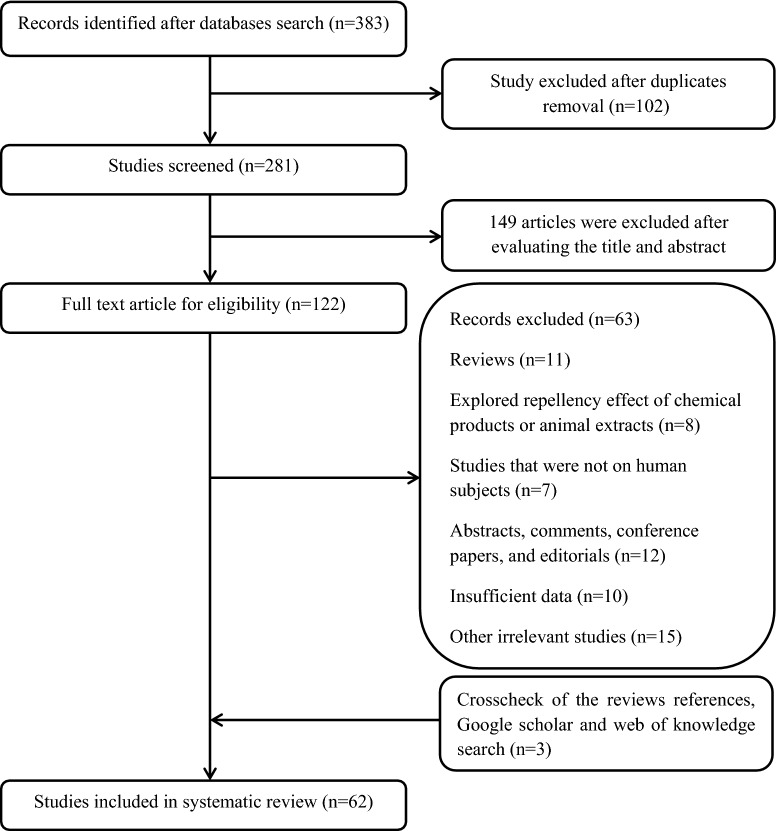
Study selection process, up to July 2018

A total of 383 studies were found by the initial literature search of the databases. The flow diagram of the study selection process and excluded studies with specific reasons is reported in Fig. 1. Of the 324 excluded citations, 102 were duplicated studies; 149 were not relevant to the repellency effect of plants on *Anopheles* spp. after screening titles/abstracts; 11 were review publications; 8 investigated the repellency impact of chemical-based repellents or animal extracts; 7 studies were conducted on laboratory animals; 12 were abstracts, conference papers, comments, and editorials; 10 studies had not reported sufficient data regarding the percentage of repellency or complete protection time; and, 15 studies were other irrelevant studies. The primary eligibility process yielded 59 documents and crosscheck of the references of reviews and other databases search provided 3 further articles [8–10]. A total of 62 studies conducted in different countries, including India [7–40], Thailand [4, 5, 41–48], Ethiopia [49–52], Kenya [53–57], Germany [6], Nigeria [1], USA [58], Tanzania [59], Brazil [60], Sudan [61], Iran [62], Cameron [63] and Ivory Coast [64] were eventually included in the systematic review based on the inclusion criteria for the effect of plant-based repellents on species of *Anopheles* mosquitoes. The included studies were published between 1999 and 2018. Expect for 6 studies which were field trial, other studies were conducted on laboratory condition. None of the studies reported the inclusion and exclusion criteria explicitly other than specifying a healthy volunteer. Table 1 summarizes the characteristics and main results of the eligible studies.

**Table 1:** Characteristics of studie**s**. Characteristics of studies included in the systematic review, up to July 2018

Study	Year	Country	Study type	Plant extract/(essential oil)	Concentration dose	*Anopheles* species	Repellency %	Protection time (hours)
Ansari et al.	2005	India	Field	Pine oil (*Pinus*)	1 ml without dilution	*An. culicifacies*	100	11
				Citronella (lemongrass oil)	1 ml without dilution	*An. culicifacies*	100	11
Ansari et al.	2000	India	Field	*D. sissoo* oil	1 ml without dilution	*An. culicifacies*	96.1	10.3
				*D. sissoo* oil	1 ml without dilution	*An. annularis*	100	11
				*D. sissoo* oil	1 ml without dilution	*An. subpictus*	89.7	8
Ansari et al.	2000	India	Field	Peppermint oil	1 ml without dilution	*An. culicifacies*	92.3	9.6
				Peppermint oil	1 ml without dilution	*An. annularis*	100	11
				Peppermint oil	1 ml without dilution	*An. subpictus*	83.1	7.3
Amer et al.	2006	Germany	Laboratory	Citronella (*Cymbopogon winterianus*) essential oils	20% oil solutions	*An. stephensi*	52.4	8
				Rosewood (*Aniba rosaeodora*) essential oils	20% oil solutions	*An. stephensi*	4.8	6.5
				Lavender (*Lavandula angustifolia*) essential oils	20% oil solutions	*An. stephensi*	80.9	8
				Camphor (*C. camphora*) essential oils	20% oil solutions	*An. stephensi*	42.8	8
				Catnip (*N. cataria*) essential oils	20% oil solutions	*An. stephensi*	100	8
				Geranium (*Pelargonium graveolens*) essential oils	20% oil solutions	*An. stephensi*	61.9	8
				Thyme (*T. serpyllum*) essential oils	20% oil solutions	*An. stephensi*	33.3	7.5
				Eucalyptus (*E. globulus*) essential oils	20% oil solutions	*An. stephensi*	28.6	5.5
				Jasmine (*Jasminum grandiflorum*) essential oils	20% oil solutions	*An. stephensi*	100	8
				Broad-leaved eucalyptus (*Eucalyptus dives*) essential oils	20% oil solutions	*An. stephensi*	38.1	8
				Lemongrass (*Cymbopogon citratus*) essential oil	20% oil solutions	*An. stephensi*	100	8
				Lemon-scented eucalyptus (*E. citriodora*) essential oil	20% oil solutions	*An. stephensi*	52.4	8
				Fichtennadel (*Picea excelsa*) essential oil	20% oil solutions	*An. stephensi*	19	3
				Amyris (*Amyris balsamifera*) essential oil	20% oil solutions	*An. stephensi*	100	8
				Lemon (*Citrus limon*) essential oil	20% oil solutions	*An. stephensi*	9.5	7
				Narrow-leaved eucalyptus (*Eucalyptus radiata*) essential oil	20% oil solutions	*An. stephensi*	42.8	8
				Carotin oil (*Glycina soja*) essential oil	20% oil solutions	*An. stephensi*	9.5	8
				Cedarwood (*Juniperus virginiana*) essential oil	20% oil solutions	*An. stephensi*	38.1	8
				frankincense (*Boswellia carteri*) essential oil	20% oil solutions	*An. stephensi*	19	5
				Dill (*Anethum graveolens*) essential oil	20% oil solutions	*An. stephensi*	71.4	3.5
				Myrtle (*M. communis*) essential oil	20% oil solutions	*An. stephensi*	42.8	6.5
				Chamomile (*Anthemis nobilis*) essential oil	20% oil solutions	*An. stephensi*	76.2	8
				Cinnamon (*C. zeylanicum*) essential oil	20% oil solutions	*An. stephensi*	100	8
				Juniper (*Juniperus communis*) essential oil	20% oil solutions	*An. stephensi*	76.2	8
				Sage (*Salvia sclarea*) essential oil	20% oil solutions	*An. stephensi*	19	5
				Peppermint (*Mentha piperita*) essential oil	20% oil solutions	*An. stephensi*	57.1	6.5
				Basil (*Ocimum basilicum*) essential oil	20% oil solutions	*An. stephensi*	66.7	3.5
				Cajeput (*Melaleuca leucadendron*) essential oil	20% oil solutions	*An. stephensi*	100	8
				Soya bean (*Glycina max*) essential oil	20% oil solutions	*An. stephensi*	76.2	8
				Rosemary (*R. officinalis*) essential oil	20% oil solutions	*An. stephensi*	100	8
				Niaouli (*Melaleuca quinquenervia*) essential oil	20% oil solutions	*An. stephensi*	100	8
				Olive (*O. europaea*) essential oil	20% oil solutions	*An. stephensi*	71.4	8
				Black pepper (*Piper nigrum*) essential oil	20% oil solutions	*An. stephensi*	61.9	3
				Verbena (*Lippia citriodora*) essential oil	20% oil solutions	*An. stephensi*	38.1	5.5
				tagetes (*T. minuta*) essential oil	20% oil solutions	*An. stephensi*	100	8
				Violet (*Viola odorata*) essential oil	20% oil solutions	*An. stephensi*	100	8
				Sandalwood (*Santalum album*) essential oil	20% oil solutions	*An. stephensi*	100	8
				Litsea (*Litsea cubeba*) Essential oil	20% oil solutions	*An. stephensi*	100	8
				*Helichrysum* (*Helichrysum italicum*) essential oil	20% oil solutions	*An. stephensi*	47.6	6
				Galbanum (*Ferula galbaniflua*) essential oil	20% oil solutions	*An. stephensi*	100	8
				Chamomile (*Chamaemelum nobile*) essential oil	20% oil solutions	*An. stephensi*	47.6	5.5
Amerasan et al.	2012	India	Laboratory	*Cassia tora* Linn methanol extract	1 mg/cm^2^ 2.5 mg/cm^2^ 5.0 mg/cm^2^	*An. stephensi*	100 100 100	2 2 2.5
Abiy et al.	2015	Ethiopia	Field	20% neem oil	Neem and chinaberry oils were diluted to 20% using Niger seed (noog abyssinia) oil	*An. arabiensis*	71	3
				20% chinaberry oil	Neem and chinaberry oils were diluted to 20% using Niger seed (noog abyssinia) oil	*An. arabiensis*	70	1
Alayo et al.	2015	Nigeria	Laboratory	*Cassia mimosoides* petroleum ether extract	Cream 0.5% w/w	*An. gambiae*	48	–
					Cream 1% w/w		88	–
					Cream 2% w/w		100	0.08
					Cream 4% w/w		100	0.08
					Cream 6% w/w		100	0.08
Alwala et al.	2010	Kenya	Laboratory	*Mangifera indica* essential Oil	10% solution	*An. gambiae*	100	–
Baskar et al.	2018	India	Laboratory	*Atalantia monophylla* essential oil	50 ppm	*An. stephensi*	–	6.85
Govindarajan et al.	2010	India	Laboratory	*Sida acuta* Burm. F. extract	2.5 mg/cm^2^	*An. stephensi*	100	2.5
					5 mg/cm^2^	*An. stephensi*	100	3
Govindarajan et al.	2011	India	Laboratory	*Ervatamia coronaria* extract	1 mg/cm^2^ 2.5 mg/cm^2^ 5 mg/cm^2^	*An. stephensi* *An. stephensi* *An. stephensi*	100 100 100	2.5 3 3.5
				*Caesalpinia pulcherrima* extract	1 mg/cm^2^	*An. stephensi*	100	2
					2.5 mg/cm^2^	*An. stephensi*	100	2.5
Govindarajan et al.	2011	India	Laboratory		5 mg/cm^2^	*An. stephensi*	100	3
					2.5 mg/cm^2^ 5 mg/cm^2^	*An. subpictus* *An. subpictus*	100 100	2 2.5
				*R. officinalis* L. essential oil	1 mg/cm^2^	*An. subpictus*	100	1
					2.5 mg/cm^2^	*An. subpictus*	100	1
					5 mg/cm^2^	*An. subpictus*	100	1.5
				*C. citrates* Stapf. essential oil	1 mg/cm^2^	*An. subpictus*	100	1
					2.5 mg/cm^2^	*An. subpictus*	100	1.5
					5 mg/cm^2^	*An. subpictus*	100	2
				*C. zeylanicum* L. essential oil	1 mg/cm^2^	*An. subpictus*	100	1
					2.5 mg/cm^2^	*An. subpictus*	100	1
					5 mg/cm^2^	*An. subpictus*	100	1.5
Govindarajan et al.	2016	India	Laboratory	*Zingiber nimmonii* essential oil	1 mg/cm^2^	*An. stephensi*	100	2
					2 mg/cm^2^	*An. stephensi*	100	2.5
					5 mg/cm^2^	*An. stephensi*	100	3
Jeyabalan et al.	2003	India	Laboratory	*P. citrosa* leaf extract	0.5%	*An. stephensi*	36	–
					1%	*An. stephensi*	51	–
					2%	*An. stephensi*	78	–
					4%	*An. stephensi*	100	–
Karunamoorthi et al.	2008	Ethiopia	Laboratory	Woira (*O. europaea*) smoke	Burning of 25 g of dried plant materials	*An. arabiensis*	79.7	–
				Tinjut (*Ostostegia integrifolia*) smoke	Burning of 25 g of dried plant materials	*An. arabiensis*	90.1	–
				Wogert (*Silene macroserene*) smoke	Burning of 25 g of dried plant materials	*An. arabiensis*	93.6	–
				Kebercho (*Echinops* sp.) extract	Burning of 25 g of dried plant materials	*An. arabiensis*	92.4	–
Karunamoorthi et al.	2010	Ethiopia	Laboratory	*C. citratus* extract	1 mg/cm^2^	*An. arabiensis*	100	3.2
					1.5 mg/cm^2^	*An. arabiensis*	100	4.4
					2 mg/cm^2^	*An. arabiensis*	100	5.3
					2.5 mg/cm^2^	*An. arabiensis*	100	6.3
Govindarajan et al.	2016	India	Laboratory	*Origanum scabrum* essential oil	1 mg/cm^2^	*An. stephensi*	100	2.5
					2 mg/cm^2^	*An. stephensi*	100	3
					5 mg/cm^2^	*An. stephensi*	100	3.5
Haldar et al.	2014	India	Laboratory	*Ficus krishnae* smoke	30 mg/l smoked	*An. stephensi*	18	0.16
					60 mg/l smoked	*An. stephensi*	100	0.5
					90 mg/l smoked	*An. stephensi*	100	1
Auysawasdi et al.	2015	Thailand	Laboratory	*Curcuma longa* essential oil	5%	*An. dirus*	100	4
					10%	*An. dirus*	100	5
					15%	*An. dirus*	100	5.5
					20%	*An. dirus*	100	5.5
					25%	*An. dirus*	100	8
				*E. globulus* essential oil	5%	*An. dirus*	100	1.7
					10%	*An. dirus*	100	2.3
					15%	*An. dirus*	100	3
					20%	*An. dirus*	100	3
					25%	*An. dirus*	100	3.4
				*Citrus aurantium* essential oil	5%	*An. dirus*	100	1.8
					10%	*An. dirus*	100	2.9
					15%	*An. dirus*	100	2.9
					20%	*An. dirus*	100	3
					25%	*An. dirus*	100	3.5
Barnard et al.	1999	USA	Laboratory	Clove essential oil	25%	*An. albimanus*	100	1.25
					50%	*An. albimanus*	100	1.5
					75%	*An. albimanus*	100	2.26
					100%	*An. albimanus*	100	3.55
				Thyme essential oil	25%	*An. albimanus*	100	0.75
					50%	*An. albimanus*	100	0.5
					75%	*An. albimanus*	100	1
					100%	*An. albimanus*	100	1.75
Kweka et al.	2008	Tanzania	Laboratory	Citronella	500 mg/m^2^	*An. gambiae*	81	–
				*Ocimum suave* extract	500 mg/m^2^	*An. gambiae*	81	–
				*Ocimum kilimandscharicum* extract	500 mg/m^2^	*An. gambiae*	73	–
				*Citronella*	500 mg/m^2^	*An. arabiensis*	85	–
				*O. suave* extract	500 mg/m^2^	*An. arabiensis*	89	–
				*O. kilimandscharicum* extract	500 mg/m^2^	*An. arabiensis*	75	–
Kovendan et al.	2012	India	Laboratory	*A. alnifolia* extract	1 mg/cm^2^	*An. stephensi*	100	2
					3 mg/cm^2^	*An. stephensi*	100	2
					5 mg/cm^2^	*An. stephensi*	100	2.5
Krishnappa et al.	2012	India	Laboratory	*A. digitata* crude extract	2 mg/cm^2^	*An. stephensi*	100	3
					4 mg/cm^2^	*An. stephensi*	100	3.5
					6 mg/cm^2^	*An. stephensi*	100	3.5
Naine et al.	2014	India	Laboratory	*Streptomyces* sp. VITJS4 extract	1 mg/cm^2^	*An. stephensi*	100	2
					3 mg/cm^2^	*An. stephensi*	100	2
					6 mg/cm^2^	*An. stephensi*	100	2
Murugan et al.	2012	India	Laboratory	Orange peel extract	50 ppm	*An. stephensi*	99	–
					150 ppm	*An. stephensi*	100	0.5
					250 ppm	*An. stephensi*	100	0.05
					350 ppm	*An. stephensi*	100	1.5
					450 ppm	*An. stephensi*	100	2
Padilha et al.	2003	Brazil	Field	*Ocimum selloi* oil	10% v/v	*An. braziliensis*	89	0.5
Konan et al.	2003	Ivory Coast	Laboratory	Karite nut butter oil	75%	*An. gambiae*	100	2
				Palm oil	75%	*An. gambiae*	100	1.38
				Coconut oil	75%	*An. gambiae*	100	0.76
Maheswaran et al.	2013	India	Laboratory	Confertifolin essential oil	0.62 ppm	*An. stephensi*	100	1
					1.25 ppm	*An. stephensi*	100	2.5
					2.5 ppm	*An. stephensi*	100	3
					5 ppm	*An. stephensi*	100	5
					10 ppm	*An. stephensi*	100	5.2
Panneerselvam et al.	2013	India	Laboratory	*Andrographis paniculata* methanol leaf extract	1 mg/cm^2^	*An. stephensi*	100	2
					3 mg/cm^2^	*An. stephensi*	100	2.5
					6 mg/cm^2^	*An. stephensi*	100	3
				*Cassia occidentalis* methanol leaf extract	1 mg/cm^2^	*An. stephensi*	100	2
					3 mg/cm^2^	*An. stephensi*	100	2.5
					6 mg/cm^2^	*An. stephensi*	100	2.5
				*Euphorbia hirta* methanol leaf extract	1 mg/cm^2^	*An. stephensi*	100	2
					3 mg/cm^2^	*An. stephensi*	100	2
					6 mg/cm^2^	*An. stephensi*	100	2.5
Panneerselvam et al.	2012	India	Laboratory	*Artemisia nilagirica* extract	50 ppm	*An. stephensi*	95	0.5
					150 ppm	*An. stephensi*	98	0.5
					250 ppm	*An. stephensi*	100	0.5
					350 ppm	*An. stephensi*	100	1
					450 ppm	*An. stephensi*	100	2
Phasomkusolsil et al.	2011	Thailand	Laboratory	*Cananga odorata* oil	0.02 mg/cm^2^	*An. dirus*	94	–
					0.10 mg/cm^2^	*An. dirus*	92	–
					0.21 mg/cm^2^	*An. dirus*	92	–
				*C. sinensis* oil	0.02 mg/cm^2^	*An. dirus*	40	–
					0.10 mg/cm^2^	*An. dirus*	54	–
					0.21 mg/cm^2^	*An. dirus*	84	–
				*C. citratus* oil	0.02 mg/cm^2^	*An. dirus*	76	–
					0.10 mg/cm^2^	*An. dirus*	82	–
					0.21 mg/cm^2^	*An. dirus*	98	–
				*Cymbopogon nardus* oil	0.02 mg/cm^2^	*An. dirus*	92	–
					0.10 mg/cm^2^	*An. dirus*	92	–
					0.21 mg/cm^2^	*An. dirus*	98	–
				*E. citriodora* oil	0.02 mg/cm^2^	*An. dirus*	52	–
					0.10 mg/cm^2^	*An. dirus*	74	–
					0.21 mg/cm^2^	*An. dirus*	86	–
				*O. basilicum* oil	0.02 mg/cm^2^	*An. dirus*	66	–
					0.10 mg/cm^2^	*An. dirus*	74	–
					0.21 mg/cm^2^	*An. dirus*	96	–
				*S. aromaticum* oil	0.02 mg/cm^2^	*An. dirus*	82	–
					0.10 mg/cm^2^	*An. dirus*	92	–
					0.21 mg/cm^2^	*An. dirus*	98	–
Prabhu et al.	2011	India	Laboratory	*Moringa oleifera* extract	20%	*An. stephensi*	23	–
					40%	*An. stephensi*	43	–
					60%	*An. stephensi*	58	–
					80%	*An. stephensi*	76	–
					100%	*An. stephensi*	90	–
Rajkumar et al.	2007	India	Laboratory	*Centella asiatica* essential oil	2%	*An. stephensi*	–	1
					4%	*An. stephensi*	–	1.78
					6%	*An. stephensi*	–	2.33
				*Ipomoea cairica* essential oil	2%	*An. stephensi*	–	2.63
					4%	*An. stephensi*	–	4.13
					6%	*An. stephensi*	–	5.53
				*Momordica charantia* essential oil	2%	*An. stephensi*	–	2.38
					4%	*An. stephensi*	–	3.93
					6%	*An. stephensi*	–	5.38
				*Psidium guajava* essential oil	2%	*An. stephensi*	–	0.93
					4%	*An. stephensi*	–	1.48
					6%	*An. stephensi*	–	1.98
				*Tridax procumbens* essential oil	2%	*An. stephensi*	–	2.33
					4%	*An. stephensi*	–	3.78
					6%	*An. stephensi*	–	5.28
Rajkumar et al.	2005	India	Laboratory	*Solanum trilobatum* extract	0.001%	*An. stephensi*	100	1.15
					0.005%	*An. stephensi*	100	1.3
					0.01%	*An. stephensi*	100	1.51
					0.015%	*An. stephensi*	100	1.7
					0.02%	*An. stephensi*	100	2.03
Rawani et al.	2012	India	Laboratory	*P. tuberosa* extract	1%	*An. stephensi*	65	2.3
					1.50%	*An. stephensi*	80	4
					2%	*An. stephensi*	90	5
Reegan et al.	2015	India	Laboratory	*Cliona celata* extract	1 mg/cm^2^	*An. stephensi*	100	1.08
					2.5 mg/cm^2^	*An. stephensi*	100	1.71
					5 mg/cm^2^	*An. stephensi*	100	1.21
Swathi et al.	2012	India	Laboratory	*Datura stramonium* extract	0.1%	*An. stephensi*	–	0.35
					0.5%	*An. stephensi*	–	0.72
					1%	*An. stephensi*	–	1.9
Seyoum et al.	2002	Kenya	Semi-field	Neem (*A. indica*)	Periodic thermal expulsion	*An. gambiae*	24.5	–
				Lemon eucalyptus (*Corymbia citriodora*)	Periodic thermal expulsion	*An. gambiae*	74.5	–
				Wild spikenard (*Hyptis suaveolens*)	Periodic thermal expulsion	*An. gambiae*	-13.3	–
				Lantana (*Lantana camara*)	Periodic thermal expulsion	*An. gambiae*	42.4	–
				Fever tea (*Lippia uckambensis*)	Periodic thermal expulsion	*An. gambiae*	45.9	–
				Lime basil (*Ocimum americanum*)	Periodic thermal expulsion	*An. gambiae*	43.1	–
				Rican blue basil (*O. kilimandscharicum*)	Periodic thermal expulsion	*An. gambiae*	52.0	–
				Tree basil (*O. suave*)	Periodic thermal expulsion	*An. gambiae*	53.1	–
				Khaki weed (*T. minuta*)	Placing branches or whole plants inside houses	*An. gambiae*	54.8	–
Sanghong et al.	2015	Thailand	Laboratory	*L. sinense* ethanolic preparations	25%	*An. minimus*	–	11.5
Das et al.	2003	India	Laboratory	*Cymbopogan martinii martinii* var *sofia* oil	1 ml without dilution	*An. sundaicus*	98	6
Nour et al.	2009	Sudan	Laboratory	Basil (*O. basilicum* L.) essential oil	0.1 ml		100	1.5
Trongtokit et al.	2005	Thailand	Laboratory	*C. nardus* essential oil	10%	*An. dirus*	–	0.66
					50%		–	0.5
					100%		–	1.16
				*P. cablin* essential oil	10%	*An. dirus*	–	1.33
					50%		–	2
					100%		–	2.83
				Mullilam (*Zanthoxylum limonella*) essential oil	10%	*An. dirus*	–	1
					50%		–	2.16
					100%		–	3.16
				Clove (*Syzygium aromaticum*) essential oil	10%	*An. dirus*	–	1.33
					50%		–	2.66
					100%		–	3.5
Yogananth et al.	2015	India	Laboratory	*R. mucronata* oil	1 mg/cm^2^	*An. stephensi*	73	7.2
					2 mg/cm^2^	*An. stephensi*	86	7.8
					3 mg/cm^2^	*An. stephensi*	92	8.5
					4 mg/cm^2^	*An. stephensi*	97	9.1
Tawatsin et al.	2000	Thailand	Laboratory	Turmeric (*C. longa*) volatile oil	3 ml	*An. dirus*	100	6
				Citronella	3 ml	*An. dirus*	100	6
				Hairy basil oil	3 ml	*An. dirus*	100	6
Singh et al.	2005	India	Laboratory	*Cyperus rotundus* Linn hexane extract	2.50%	*An. stephensi*	95	–
					5%	*An. stephensi*	99	–
					10%	*An. stephensi*	100	6
Mayeku et al.	2013	Kenya	laboratory	*Conyza newii* essential oil	0.01 g/ml	*An. gambiae*	38	–
					0.1 g/ml	*An. gambiae*	68	–
					1 g/ml	*An. gambiae*	100	–
Phasomkusolsil et al.	2009	Thailand	Laboratory	Phlai (*Z. cassumunar*) oil	100 μl	*An. minimus*	–	2
				Turmeric (*C. longa*) oil	100 μl	*An. minimus*	–	1
				Mah-Khwuaen (*Z. limonella*) oil	100 μl	*An. minimus*	–	0.66
				Citronella grass (*C. nardus*) oil	100 μl	*An. minimus*	–	2.16
				Orange oil (*Citrus sinensis*) oil	100 μl	*An. minimus*	–	0.83
				Eucalyptus (*E. citriodora*) oil	100 μl	*An. minimus*	–	0.5
				Clove (*S. aromaticum*) oil	100 μl	*An. minimus*	–	2
Trongtokit et al.	2004	Thailand	Laboratory	Clove oil	20% gel	*An. dirus*	–	4.5
					cream 20%	*An. dirus*	–	4.8
Birkett et al.	2011	Kenya	Laboratory	*N. cataria*	0.01 mg	*An. gambiae*	17	–
					0.1 mg	*An. gambiae*	97	–
					1 mg	*An. gambiae*	100	–
Kamaraj et al.	2011	India	Laboratory	*A. concinna* extract	500 ppm	*An. stephensi*	21	–
Solomon et al.	2012	Ethiopia	Laboratory	Citronella extract	20%	*An. Arabiensis*	73	–
Soonwera et al.	2015	Thailand	Laboratory	*C. odorata* oil	1%	*An. dirus*	92	–
					5%	*An. dirus*	92	–
					10%	*An. dirus*	94	–
Sritabutra et al.	2011	Thailand	Laboratory	Eucalyptus (*E. globules*) essencial oil	0.1 ml	*An. dirus*	–	1.58
				Peppermint (*M. piperita*) essencial oil	0.1 ml	*An. dirus*	–	1.08
				Garlic (*A. sativum*) essencial oil	0.1 ml	*An. dirus*	–	0.68
				Orange (*C. sinensis*) essencial oil	0.1 ml	*An. dirus*	–	0.83
				Citronella grass (*C. nardus*) essencial oil	0.1 ml	*An. dirus*	–	0.8
				Lemongrass (*C. citratus*) essencial oil	0.1 ml	*An. dirus*	–	1.63
				Clove (*S. aromaticum*) essencial oil	0.1 ml	*An. dirus*	–	1
				Sweet basil (*O. basilicum*) essencial oil	0.1 ml	*An. dirus*	–	0.75
Tavassoli et al.	2001	iran	Laboratory	Marigold (*Calendula officinalis*) essential oil	50%	*An. stephensi*	–	2.15
				Myrtle essential oil	50%	*An. stephensi*	–	4.36
Younoussa et al.	2016	Cameroon	Laboratory	*Annona senegalensis* leaf extract	4.0 mg/cm^2^	*An. gambiae*	–	0.5
					8.0 mg/cm^2^	*An. gambiae*	–	1
					12.0 mg/cm^2^	*An. gambiae*	–	1.5
				*Boswellia dalzielii* leaf extract	4.0 mg/cm^2^	*An. gambiae*	46	
					8.0 mg/cm^2^	*An. gambiae*	–	0.5
					12.0 mg/cm^2^	*An. gambiae*	–	1
Govindarajan et al.	2011	India	Laboratory	*Coccinia indica* extract	1 mg/cm^2^	*An. stephensi*	100	3
					2.5 mg/cm^2^	*An. stephensi*	100	3
					5 mg/cm^2^	*An. stephensi*	100	3.5
Govindarajan et al.	2012	India	Laboratory	*Cardiospermum halicacabum* oil	1 mg/cm^2^	*An. stephensi*	100	2
					2.5 mg/cm^2^	*An. stephensi*	100	2.5
					5 mg/cm^2^	*An. stephensi*	100	3
Govindarajan et al.	2014	India	Laboratory	*Asparagus racemosus* crude extract	1 mg/cm^2^	*An. stephensi*	100	2.5
					2 mg/cm^2^	*An. stephensi*	100	2.5
					5 mg/cm^2^	*An. stephensi*	100	3
Govindarajan et al.	2015	India	Laboratory	*Delonix elata* crude extract	1 mg/cm^2^	*An. stephensi*	100	2.5
					2.5 mg/cm^2^	*An. stephensi*	100	3
					5 mg/cm^2^	*An. stephensi*	100	3.5
Innocent et al.	2014	Kenya	Laboratory	*Uvariodendron gorgonis* essential oil	0.01 w/v	*An. gambiae*	29	–
					0.1 w/v	*An. gambiae*	48	–
					1 w/v	*An. gambiae*	57	–
					10 w/v	*An. gambiae*	64	–
				*Clausena anisata* essential oil	0.01 w/v	*An. gambiae*	13	–
					0.1 w/v	*An. gambiae*	21	–
					1 w/v	*An. gambiae*	42	–
					10 w/v	*An. gambiae*	56	–
				*Lantana vibunoides* essential oil	0.01 w/v	*An. gambiae*	26	–
					0.1 w/v	*An. gambiae*	46	–
					1 w/v	*An. gambiae*	54	–
					10 w/v	*An. gambiae*	62	–
Kumar et al.	2012	India	Laboratory	*Sargassum wightii* Greville methanolic extract	2 mg/l	*An. sundaicus*	26	–
					4 mg/l	*An. sundaicus*	40	–
					6 mg/l	*An. sundaicus*	57	–
					8 mg/l	*An. sundaicus*	71	–
					10 mg/l	*An. sundaicus*	89	–
Madhiyazhagan et al.	2014	India	Laboratory	*O. canum* extract	0.49 mg/l	*An. stephensi*	63	–
					0.99 mg/l	*An. stephensi*	77	–
					1.99 mg/l	*An. stephensi*	86	–

### Effectiveness of plant-based products against *Anopheles* spp.

Potential plant-based repellents stratified by protection time with at least 4 h protection time are reported in Table 2. The highest repellency effect was identified from *Ligusticum sinense* extract, followed by citronella, pine, *Dalbergia sissoo*, peppermint and *Rhizophora mucronata* oils with complete protection time ranging from 9.1 to 11.5 h. Ethanolic 25% extract of *L. sinense* was able to completely repel *Anopheles minimus* for 11.5 h. Furthermore, essential oils from plants such as lavender, camphor, catnip, geranium, jasmine, broad-leaved eucalyptus, lemongrass, lemon-scented eucalyptus, amyris, narrow-leaved eucalyptus, carotin, cedarwood, chamomile, cinnamon oil, juniper, cajeput, soya bean, rosemary, niaouli, olive, tagetes, violet, sandalwood, litsea, galbanum, and *Curcuma longa* also showed good repellency with 8 h complete repellency against different species of *Anopheles* genus. Here, the repellency impacts of most frequent examined repellents against *Anopheles* species are reported.

**Table 2:** Stratification of potential of plant based repellents

Protection time (hours)	Plant name	Concentration/dose	Anopheles species
11.5	*L. sinense* ethanolic extract	25%	*An. minimus*
11	Pine oil (*Pinus*) Citronella (lemongrass oil) *D. sissoo* oil Peppermint oil	1 ml without dilution 1 ml without dilution 1 ml without dilution 1 ml without dilution	*An. culicifacies* *An. culicifacies* *An. annularis* *An. annularis*
8 < to < 10	*D. sissoo* oil Peppermint oil *R. mucronata* oil *R. mucronata* oil	1 ml without dilution 1 ml without dilution 4 mg/cm^2^ 3 mg/cm^2^	*An. culicifacies* *An. culicifacies* *An. stephensi* *An. stephensi*
8	*D. sissoo* oil	1 ml without dilution	*An. subpictus*
	Citronella (*C. winterianus*) essential oils	20% oil solution	*An. stephensi*
	Lavender (*L. angustifolia*) essential oils	20% oil solution	*An. stephensi*
	Camphor (*C. camphora*) essential oils	20% oil solution	*An. stephensi*
	Catnip (*N. cataria*) essential oils	20% oil solution	*An. stephensi*
	Geranium (*P. graveolens*) essential oils	20% oil solution	*An. stephensi*
	Jasmine (*J. grandiflorum*) essential oils	20% oil solution	*An. stephensi*
	Broad-leaved eucalyptus (*E. dives*) essential oils	20% oil solution	*An. stephensi*
	Lemongrass (*C. citratus*) essential oil	20% oil solution	*An. stephensi*
	Lemon-scented eucalyptus (*E. citriodora*)	20% oil solution	*An. stephensi*
	Amyris (*A. balsamifera*) essential oil	20% oil solution	*An. stephensi*
	Narrow-leaved eucalyptus (*E. radiata*) essential oil	20% oil solution	*An. stephensi*
	Carotin oil (*G. soja*) essential oil	20% oil solution	*An. stephensi*
	Cedarwood (*J. virginiana*) essential oil	20% oil solution	*An. stephensi*
	Chamomile (*A. nobilis*) essential oil	20% oil solution	*An. stephensi*
	Cinnamon (*C. zeylanicum*) essential oil	20% oil solution	*An. stephensi*
	Juniper (*J. communis*) essential oil	20% oil solution	*An. stephensi*
	Cajeput (*M. leucadendron*) essential oil	20% oil solution	*An. stephensi*
	Soya bean (*G. max*) essential oil	20% oil solution	*An. stephensi*
	Rosemary (*R. officinalis*) essential oil	20% oil solution	*An. stephensi*
	Niaouli (*M. quinquenervia*) essential oil	20% oil solution	*An. stephensi*
	Olive (*O. europaea*) essential oil	20% oil solution	*An. stephensi*
	Tagetes (*T. minuta*) essential oil	20% oil solution	*An. stephensi*
	Violet (*V. odorata*) essential oil	20% oil solution	*An. stephensi*
	Sandalwood (*S. album*) essential oil	20% oil solution	*An. stephensi*
	Litsea (*L. cubeba*) essential oil	20% oil solution	*An. stephensi*
	Galbanum (*F. galbaniflua*) essential oil	20% oil solution	*An. stephensi*
	*C. longa* essential oil	25%	*An. dirus*
7 < to < 8	*R. mucronata* oil	2 mg/cm^2^	*An. stephensi*
	Thyme (*T. serpyllum*) essential oils	20% oil solutions	*An. stephensi*
	Peppermint oil	1 ml without dilution	*An. subpictus*
	*R. mucronata* oil	1 mg/cm^2^	*An. stephensi*
7	Lemon (*C. limon*) essential oil	20% oil solution	*An. stephensi*
6 < to < 7	*A. monophylla* essential oil	50 ppm	*An. stephensi*
	rosewood (*A. rosaeodora*) essential oils	20% oil solution	*An. stephensi*
	myrtle (*M. communis*) essential oil	20% oil solution	*An. stephensi*
	peppermint (*M. piperita*) essential oil	20% oil solution	*An. stephensi*
6	*Helichrysum* (*H. italicum*) essential oil	20% oil solution	*An. stephensi*
	*C. martinii martinii* var *sofia* oil	1 ml without dilution	*An. sundaicus*
	Turmeric (*C. longa*) volatile oil	3 ml	*An. dirus*
	Citronella	3 ml	*An. dirus*
	Hairy basil oil	3 ml	*An. dirus*
	*C. rotundus* Linn hexane extract	10%	*An. stephensi*
5 < to < 6	*I. cairica* essential oil	6%	*An. stephensi*
	Eucalyptus (*E. globulus*) essential oils	20% oil solution	*An. stephensi*
	Verbena (*L. citriodora*) essential oil	20% oil solution	*An. stephensi*
	Chamomile (*C. nobile*) essential oil	20% oil solution	*An. stephensi*
	*C. longa* essential oil	15%	*An. dirus*
	*C. longa* essential oil	20%	*An. dirus*
	*M. charantia* essential oil	6%	*An. stephensi*
	*C*. *citratus* extract	2 mg/cm^2^	*An. arabiensis*
	*T. procumbens* essential oil	6%	*An. stephensI*
	Confertifolin essential oil	10 ppm	*An. stephensi*
5	Frankincense (*B. carteri*) essential oil	20% oil solution	*An. stephensi*
	Sage (*S. sclarea*) essential oil	20% oil solution	*An. stephensi*
	*C. longa* essential oil	10%	*An. dirus*
	Confertifolin essential oil *P. tuberosa* extract	5 ppm 2%	*An. stephensi* *An. stephensi*
4 < to < 5	Clove oil Clove oil *C. citratus* extract Myrtle essential oil *I. cairica* essential oil	Cream 20% 20% gel 1/5 mg/cm^2^ 50% 4%	*An. dirus* *An. dirus* *An. arabiensis* *An. stephensi* *An. stephensi*
4	*C. longa* essential oil	5%	*An. dirus*
	*P. tuberosa* extract	1.5%	*An. stephensi*

Stratification of potential of plant based repellents by complete protection times, up to July 2018

### Citronella

The repellency effect of citronella was investigated in several studies. Citronella is an essential oil extracted from the stems and leaves of different species of lemongrass (*Cymbopogon* spp.) [65]. Ansari et al. [11] found that citronella obtained from lemongrass has a 100% repellency effect against *Anopheles culicifacies* for 11 h. Amer et al. [6] and Tawatsin et al. [44] also reported that citronella could repel *Anopheles stephensi* and *Anopheles dirus* for 8 and 6 h, respectively. Moreover, 100 μl and 0.1 ml of citronella grass essential oil showed 2.16 and 0.8 h complete protection time against *An. minimus* [45] and *An. dirus* [47], respectively. The percentage repellency of citronella in other studies. [6, 52, 59], depending on the concentration of extracts and *Anopheles* species, was reported to be 52 to 85%.

### Peppermint

Peppermint is a hybrid mint from cross-breeding spearmint (*Mentha spicata*) and water mint (*Mentha aquatica*), which contains biologically active constituents and has high menthone, menthol and methyl esters. The plant, indigenous to Europe, is now widespread in cultivation worldwide [66]. The effect of peppermint on *Anopheles* was explored in 3 studies. Ansari et al. [12] in a field trial revealed that 1 ml peppermint oil without dilution completely repels *Anopheles annularis*, *An. culicifacies* and *Anopheles subpictus* for 11, 9.6 and 7.3 h, respectively and the corresponding percentage repellency were 100%, 92.3% and 83.1%. In another study [6], 20% oil solutions of peppermint had 57% repellency and complete protection time for 6.5 h against *An. stephensi.* The study by Sritabutra et al. [47] also found that 0.1 ml of peppermint essential oil protect against *An. dirus* for 1.08 h.

### Cinnamomum

*Cinnamomum* is a genus in the Laurel family, Lauraceae, several of which are investigated for their antibacterial activity by means of essential oils from bark and leaves [67]. Amer et al. [6] reported that 20% oil solutions of both camphor (*Cinnamomum camphora*) and cinnamon (*Cinnamomum zeylanicum*) had 100% repellency affect against *An. stephensi*. While, in the study conducted by Govindarajan et al. [22], *C. zeylanicum* at 1 mg/cm^2^ showed 1 h protection against *An. subpictus*.

### Catnip (*Nepeta cataria*)

Catnip is a perennial plant that belongs to the mint family, Labiatae. This herb is spread from central Europe to central Asia and the Iranian plateaus [68]. The 20% oil solution of catnip in the study carried out by Amer et al. [6], with 100% protection against *An. stephensi* for 8 h, had a good effectiveness in preventing *Anopheles* mosquitoes. Nevertheless, Birkett et al. [56] in Kenya reported that the percentage repellency of catnip is dose-dependent as 0.01 mg, 0.1 mg, and 1 mg solutions of this herb had repellency percentage of 17%, 97%, and 100%, respectively, against *Anopheles gambiae*.

### Thyme (*Thymus serpyllum*)

Thyme is one of nine species belonging to *T. serpyllum*, a perennial aromatic plant of the Mediterranean flora [69]. *Thymus* species have been reported to possess various beneficial effects, such as antiseptic, carminative, antimicrobial, and antioxidant properties [70]. The 20% oil solution of thyme in the study conducted by Amer et al. [6], with 100% protection against *An. stephensi* for 7.5 h, had a good effectiveness in preventing *Anopheles* mosquitoes. Nevertheless, another study [58] reported that the complete protection time of thyme at its maximum concentration (100%) is 1.7 h against *Anopheles albimanus*.

### Olive (*Olea europaea*)

Olive (*O. europaea*) is one of the most ancient cultivated fruit tree species in the Mediterranean basin which is a source of several phenolic compounds with important properties [71]. The 20% oil solution of olive in the study conducted by Amer et al. [6], with a mean percentage of repellency (71.4%) and complete protection time against *An. stephensi* for 8 h, had a good effectiveness in preventing *An. stephensi* mosquitoes. Karunamoorthi et al. [50] also supported that burning of 25 g of dried *O. europaea*, comparable to Amer et al. [6], has a percentage repellency of 79.7 against *Anopheles arabiensis*.

### Eucalyptus

Eucalyptus is a significant short rotation pulpy woody plant, grown generally in tropical regions [72]. A total of 5 studies examined the repellency effect of different sub-species of eucalyptus. In the laboratory trial by Amer et al. [6], narrow-leaved eucalyptus, lemon-scented eucalyptus, and broad-leaved eucalyptus protected against *An. stephensi* for 8 h, while *Eucalyptus globulus* complete protection time was reported to be 5.5 h. Auysawasdi et al. [41] used *E. globulus* essential oil at 5%, 10%, 15%, 20% and 25% concentrations against *An. dirus*. All concentrations of *E. globulus* provided complete repellency ranging from 1.7 to 3.4 h, depending on the concentration applied. *Eucalyptus globulus* at 0.1 ml dose in a study [47] repelled *An. dirus* for 1.58 h. Besides, 100 μl *Eucalyptus citriodora* repelled *An. minimus* for 0.5 h [45]. In contrast, Seyoum et al. found that lemon eucalyptus extract is not affective against *An. gambiae* [54].

### Myrtle (*Myrtus communis*)

Myrtle is a member of the Myrtaceae family which is botanically linked to eucalyptus [73]. In 2 studies, repellency effectiveness of myrtle was investigated. The 20% oil solution of myrtle in the study conducted by Amer et al. [6], with mean percentage repellency of 42.8% and complete protection time against *An. stephensi* for 6.5 h, had a good effectiveness in preventing *Anopheles* mosquitoes. Tavassoli et al. [62] also supported that myrtle at 50% concentration repels *An. stephensi* for 4.36 h.

### Basil

Basil is an annual plant of the *Ocimum* genus, which belongs to the Lamiaceae family and is used in traditional medicine in many parts of the world [74]. In 6 studies, repellency effectiveness of basil against different *Anopheles* species was investigated. In the laboratory trial by Amer et al. [6], 20% oil solution of basil essential oil, with mean percentage repellency of 66.7%, had 100% protective impact against *An. stephensi* for 3.5 h. Phasomkusolsil et al. [42] used basil essential oil at 0.02, 0.10, and 0.21 mg/cm^2^ concentrations against *An. dirus*. The percentage repellency was dose–response and was reported to be 66%, 74% and 96%, respectively. Basil at 0.1 ml dose in other studies [47, 61] repelled *Anopheles* for 1.5 h and 0.75 h, whereas, Tawatsin et al. [44] found that hairy basil oil provides 100% protection against *An. dirus* for 6 h. In contrast, in the study by Seyoum et al. [54], no remarkable repellency effect against *An. gambiae* was identified.

### Tagetes (*Tagetes minuta*)

*Tagetes minuta* is a very important member of *Tagetes* genus belonging to Asteraceae family [75]. In 2 studies, repellency effectiveness of tagetes was explored. The 20% oil solution of *T. minuta* in the study conducted by Amer et al. [6], with complete protection time for 8 h, had a good effectiveness in preventing against *An. stephensi*. In contrast, Seyoum et al. found that tagetes extract is not affective against *An. gambiae* [54].

### Neem (*Azadirachta indica*)

Neem is a versatile tree broadly grown in tropical areas of India [76]. The repellency effect of Neem against different species of *Anopheles* was investigated in 2 studies. The 20% Neem oil in a field trial conducted by Amer et al. [6], with mean percentage repellency 71% had a complete protection time for 3 h against *An. arabiensis*. Nevertheless, Seyoum et al. found that Neem extract is not affective against *An. gambiae* [54].

### Rosemary (*Rosmarinus officinalis*)

Rosemary is an evergreen aromatic shrub with a Mediterranean origin, which belongs to Lamiaceae (Labiatae) family [77]. In 2 studies, repellency effectiveness of rosemary was reported. The 20% oil solution of rosemary in the study conducted by Amer et al. [6], with 100% protection against *An. stephensi* for 8 h, had a good effectiveness in preventing *Anopheles* mosquitoes. Govindarajan et al. [22] also supported that rosemary at 1, 2.5 and 5 mg/cm^2^ concentrations completely repels *An. subpictus* for 1, 1, and 1.5 h, respectively.

### Clove (*Syzygium aromaticum*)

Clove is a naturally occurring spice which has been shown to possess anti-bacterial, anti-oxidant, anti-pyretic, anti-candidal, and aphrodisiac activities [78]. The repellency effect of clove against different species of *Anopheles* was investigated in 6 studies. In the study by Phasomkusolsil et al. [42], clove at 0.02, 0.10 and 0.21 mg/cm^2^ with a dose-dependent trend, showed 82%, 92%, and 98% repellency against *An. dirus*. Barnard et al. [58] used clove essential oil at 25%, 50%, 75%, and 100% concentrations against *An. albimanus* and found that all concentrations of clove provided complete repellency ranging from 1.25 to 3.55 h, depending on the concentration applied. Consistently, clove at 10%, 50%, and 100% concentrations, with a dose-dependent trend, showed 1.33, 2.66, and 3.5 h complete repellency against *An. dirus* [43]. *Anopheles dirus* was repelled by clove for 1 h in laboratory conditions in Thailand [47]. Another study [45] reported that clove repels *An. minimus* for 2 h. Moreover, 20% gel of clove protected against *An. dirus* for 4.5 h [46]. All these findings support that clove can be a considered as moderate repellent.

### Orange oil (*Citrus sinensis*)

Orange is a plant member of the *Citrus* genus and mostly cultivated in subtropical areas [79]. The repellency effect of orange against different species of *Anopheles* was investigated in 4 studies. In the study by Murugan et al. [27], orange extract at 50, 150 and 250, 350, and 450 ppm showed 0, 0.5, 0.5, 1.5 and 2 h complete protection time repellency (100%) against *An. stephensi*, respectively. While, in another study [45], it repelled *An. minimus* for 0.83 h. Similarly, Sritabutra et al. [47] showed that orange repels *An. dirus* for 0.83 h. Phasomkusolsi et al. [42] also found that orange at 0.02, 0.10, and 0.21 mg/cm^2^, with a dose-dependent trend, has 44%, 54%, and 84% repellency against *An. dirus*, respectively.

### Turmeric (*C. longa*)

The medicinal plant turmeric, which is a perennial herb, and a member of Zingiberacae family, is commonly used as a spice in human food [80]. In 3 studies, repellency effectiveness of turmeric was examined. Auysawasdi et al. [41] used turmeric essential oil at 5%, 10%, 15%, 20%, and 25% concentrations against *An. dirus*. All concentrations of turmeric, with a dose–response manner, provided complete repellency ranging from 4 to 8 h, depending on the concentration applied. Other studies also found that turmeric oil repels *An. dirus* for 6 h [44] and *An. minimus* [45] for 1 h.

## Discussion

A high level of insecticide resistance has made because of the chemical control of the pests and vectors. To overcome this problem, it is essential to research for alternative approaches to vector control. The field of herbal repellents is extremely fertile as people demand mosquitoes’ repellents that are safe, pleasant to usage and ecologically maintainable. As cost is a significant factor, examination of the use of local florae as repellents is highly suggested. Essential oils and extracts of plants are emerging as potential agents for *Anopheles* spp. control, with easy-to-administer, low-cost, and risk-free properties. In the present systematic review the highest repellency effect against *Anopheles* mosquitoes was found from *L. sinense* extract, followed by citronella, pine, *D. sissoo*, peppermint and *R. mucronata* oils with complete protection time ranging from 9.1 to 11.5 h. Essential oils from plants such as lavender, camphor, catnip, geranium, jasmine, broad-leaved eucalyptus, lemongrass, lemon-scented eucalyptus, amyris, narrow-leaved eucalyptus, carotin, cedarwood, chamomile, cinnamon oil, juniper, cajeput, soya bean, rosemary, niaouli, olive, tagetes, violet, sandalwood, litsea, galbanum, and *C. longa* also showed good repellency with 8 h complete repellency against different species of *Anopheles* genus.

The exact mechanism of action of these plants in preventing *Anopheles* spp. bites has not yet been completely clarified. For citronella, as one of the most explored plant for repellency effect against various mosquitoes, it is reported that active compounds in citronella extract for repelling mosquitoes are eugenol, eucalyptol, camphor, linalool, citral, and citronellal [81]. Some data proposes that these agents interfere with olfactory receptors of mosquitoes [82]. A recent study revealed that *An. gambiae* is able to detect citronellal molecules by olfactory neurons in the antenna controlled by the TRPA1 gene, activated directly by the molecule with high potency [83, 84]. Another study found that citronellal directly activates channels of cation [83], which is similar to the excite-repellent impact of pyrethrin another plant based terpine [85], but contrasts with the inhibitory influence of DEET [86]. Although the protection time of citronella oil is shorter than that of DEET. Citronella oil could provide sufficient protection time against mosquitoes. For other plants, the underlying mechanism remains to be elucidated. Possibly, the most important aspect in increasing the permanence of such repellents that are effective but volatile is improving formulations of plant extracts to elevate their longevity through the development of nanoemulsions, improved formulations, and fixatives. While alternative uses such as excite-repellency and spatial activity have also been examined [87].

Some caution is important when interpreting the findings. First, a poorly inspected confounding aspect is the effect of sweating on the effectiveness and protection time of repellents, which are approximately all water-soluble, and this might limits the comparability of repellents. Second, in field trial studies, the number of human volunteers as well as the season during which the trial had been performed differed among the included studies. Climate could also affect mosquito behaviour and the variance is controlled by standardizing humidity temperature in ‘arm-in-cage’ trials; however, these parameters are not always similar in different trials or conform to the mosquito environment standards. Third, it should be highlighted that some plant compounds are irritating to the skin and/or highly toxic to mammals, and natural does not equate to safe. Thus, plants with potential repellency properties should be tested for their possible unpleasant side effects before introducing as alternative products. Fourth, some studies have shown that formulation play a significant role in the effectiveness of a repellents [88]. However, studies have focused more on the search for active compounds than on optimal formulations [8, 29]. Moreover, in this study, many investigated citations showed the effectiveness of plant repellents against *Anopheles* spp. mosquitoes. However, when focusing on *Anopheles* subspecies, there were only a few publications indicating the efficacy of each plant, which resulted in a difficulty to reach a robust conclusion regarding the best herbal candidates to develop new commercial repellents.

This is another area for additional research. Finally, current studies are difficult to be compared and the repellency effectiveness may also differ among subspecies. Unfortunately, a few studies aimed to compare repellency efficacy of a special plant on subspecies of *Anopheles*. The heterogeneity in the results of the previous studies might be stem from differences in compound concentrations, application dosages, mosquito species, formulations and the assessment method of repellency, as in some trials the protection time until mosquitoes landed was recorded, whereas in the majority of studies the time until mosquitoes bite was considered. Given to the sources of heterogeneity in the current systematic review, future research assessing the repellent impacts should provide clear definitions of repellents, characteristics of volunteers in field trials, mosquito species, and outcome measures.

## Conclusion

The results of this study showed that some plants essential oils and extracts have significant repellent activity against *Anopheles* spp. mosquitoes. The studies in the last two decades have focused on the search for new natural repellents and some plants displayed good repellent activities, but few natural products have been developed so far [88, 89]. This review calls for the attention of entomologists and people in the field of mosquito-transmitted diseases for understanding the value and potential position of the plant-derived repellents and their role in disease control.

## Data Availability

All data generated or analysed during this study are included in this published article.

## Abbreviation

DEET: N, N-diethyl-meta-toluamide

## References

[1] Alayo M, Femi-Oyewo M, Bakre L, Fashina A (2015). Larvicidal potential and mosquito repellent activity of Cassia mimosoides extracts. Southeast Asian J Trop Med Public Health.

[2] Karunamoorthi K, Girmay A, Hayleeyesus SF (2014). Mosquito repellent activity of essential oil of Ethiopian ethnomedicinal plant against Afro-tropical malarial vector *Anopheles arabiensis*. J King Saud Univ Sci..

[3] Karunamoorthi K (2014). The counterfeit anti-malarial is a crime against humanity: a systematic review of the scientific evidence. Malar J..

[4] Sanghong R, Junkum A, Chaithong U, Jitpakdi A, Riyong D, Tuetun B (2015). Remarkable repellency of *Ligusticum sinense* (Umbelliferae), a herbal alternative against laboratory populations of *Anopheles minimus* and *Aedes aegypti* (Diptera: Culicidae). Malar J..

[5] Soonwera M (2015). Efficacy of essential oil from Cananga odorata (Lamk.) Hook. f. & Thomson (Annonaceae) against three mosquito species *Aedes aegypti* (L.), *Anopheles dirus* (Peyton and Harrison), and *Culex quinquefasciatus* (Say). Parasitol Res.

[6] Amer A, Mehlhorn H (2006). Repellency effect of forty-one essential oils against Aedes, Anopheles, and Culex mosquitoes. Parasitol Res.

[7] Govindarajan M, Rajeswary M, Arivoli S, Tennyson S, Benelli G (2016). Larvicidal and repellent potential of *Zingiber nimmonii* (J. Graham) Dalzell (Zingiberaceae) essential oil: an eco-friendly tool against malaria, dengue, and lymphatic filariasis mosquito vectors?. Parasitol Res.

[8] Panneerselvam C, Murugan K (2013). Adulticidal, repellent, and ovicidal properties of indigenous plant extracts against the malarial vector, *Anopheles stephensi* (Diptera: Culicidae). Parasitol Res.

[9] Govindarajan M, Sivakumar R (2012). Repellent properties of *Cardiospermum halicacabum* Linn (Family: Sapindaceae) plant leaf extracts against three important vector mosquitoes. Asian Pac J Trop Biomed..

[10] Govindarajan M, Mathivanan T, Elumalai K, Krishnappa K, Anandan A (2011). Ovicidal and repellent activities of botanical extracts against *Culex quinquefasciatus*, *Aedes aegypti* and *Anopheles stephensi* (Diptera: Culicidae). Asian Pac J Trop Biomed..

[11] Ansari M, Mittal P, Razdan R, Sreehari U (2005). Larvicidal and mosquito repellent activities of pine (*Pinus longifolia*, Family: Pinaceae) oil. J Vector Borne Dis..

[12] Ansari M, Vasudevan P, Tandon M, Razdan R (2000). Larvicidal and mosquito repellent action of peppermint (*Mentha piperita*) oil. Bioresource Technol..

[13] Ansari M, Razdan R, Tandon M, Vasudevan P (2000). Larvicidal and repellent actions of *Dalbergia sissoo* Roxb (F Leguminosae) oil against mosquitoes. Bioresource Technol..

[14] Amerasan D, Murugan K, Kovendan K, Kumar PM, Panneerselvam C, Subramaniam J (2012). Adulticidal and repellent properties of *Cassia tora* Linn. (Family: Caesalpinaceae) against *Culex quinquefasciatus*, *Aedes aegypti*, and *Anopheles stephensi*. Parasitol Res.

[15] Baskar K, Sudha V, Nattudurai G, Ignacimuthu S, Duraipandiyan V, Jayakumar M (2018). Larvicidal and repellent activity of the essential oil from *Atalantia monophylla* on three mosquito vectors of public health importance, with limited impact on non-target zebra fish. Phys Mol Plant Pathol..

[16] Govindarajan M (2010). Larvicidal and repellent activities of *Sida acuta* Burm. F. (Family: Malvaceae) against three important vector mosquitoes. Asian Pac J Trop Med..

[17] Govindarajan M (2011). Ovicidal and repellent properties of *Coccinia indica* Wight and Arn (Family: Cucurbitaceae) against three important vector mosquitoes. Eur Rev Med Pharmacol Sci..

[18] Govindarajan M, Kadaikunnan S, Alharbi NS, Benelli G (2016). Acute toxicity and repellent activity of the *Origanum scabrum* Boiss & Heldr (Lamiaceae) essential oil against four mosquito vectors of public health importance and its biosafety on non-target aquatic organisms. Enviro Sci Pollut Res..

[19] Jeyabalan D, Arul N, Thangamathi P (2003). Studies on effects of *Pelargonium citrosa* leaf extracts on malarial vector. Anopheles stephensi Liston. Bioresource Technol..

[20] Govindarajan M, Rajeswary M, Sivakumar R (2015). Repellent properties of *Delonix elata* (L) Gamble (Family: Fabaceae) against malaria vector *Anopheles stephensi* (Liston)(Diptera: Culicidae). J Saudi Soc Agric Sci..

[21] Govindarajan M, Sivakumar R (2015). Laboratory evaluation of Indian medicinal plants as repellents against malaria, dengue, and filariasis vector mosquitoes. Parasitol Res..

[22] Govindarajan M (2011). Larvicidal and repellent properties of some essential oils against *Culex tritaeniorhynchus* Giles and *Anopheles subpictus* Grassi (Diptera: Culicidae). Asian Pac J Trop Med..

[23] Haldar KM, Ghosh P, Chandra G (2014). Larvicidal, adulticidal, repellency and smoke toxic efficacy of *Ficus krishnae* against *Anopheles stephensi* Liston and *Culex vishnui* group mosquitoes. Asian Pac J Trop Dis..

[24] Kovendan K, Murugan K, Kumar PM, Thiyagarajan P, William SJ (2013). Ovicidal, repellent, adulticidal and field evaluations of plant extract against dengue, malaria and filarial vectors. Parasitol Res..

[25] Krishnappa K, Elumalai K, Dhanasekaran S, Gokulakrishnan J (2012). Larvicidal and repellent properties of *Adansonia digitata* against medically important human malarial vector mosquito *Anopheles stephensi* (Diptera: Culicidae). J Vector Borne Dis..

[26] Naine SJ, Devi S (2014). Larvicidal and repellent properties of Streptomyces sp. VITJS4 crude extract against *Anopheles stephensi*, *Aedes aegypti* and *Culex quinquefasciatus* (Diptera: Culicidae). Pol J Microbiol..

[27] Murugan K, Kumar PM, Kovendan K, Amerasan D, Subrmaniam J, Hwang J-S (2012). Larvicidal, pupicidal, repellent and adulticidal activity of *Citrus sinensis* orange peel extract against *Anopheles stephensi*, *Aedes aegypti* and *Culex quinquefasciatus* (Diptera: Culicidae). Parasitol Res..

[28] Maheswaran R, Ignacimuthu S (2013). Bioefficacy of essential oil from *Polygonum hydropipe*r L. against mosquitoes, *Anopheles stephensi* and *Culex quinquefasciatus*. Ecotoxicol Environ Saf..

[29] Panneerselvam C, Murugan K, Kovendan K, Kumar PM (2012). Mosquito larvicidal, pupicidal, adulticidal, and repellent activity of *Artemisia nilagirica* (Family: Compositae) against *Anopheles stephensi* and *Aedes aegypti*. Parasitol Res..

[30] Prabhu K, Murugan K, Nareshkumar A, Ramasubramanian N, Bragadeeswaran S (2011). Larvicidal and repellent potential of *Moringa oleifera* against malarial vector, *Anopheles stephensi* Liston (Insecta: Diptera: Culicidae). Asian Pac J Trop Biomed..

[31] Almehmadi RM (2008). Oviposition deterrent and skin repellent activities of *Artemisia herba alba*, *Matricharia chamomella* and *Melia azedarach* against *Culex quinquefasciatus*. Saudi J Biol Sci..

[32] Rajkumar S, Jebanesan A (2007). Repellent activity of selected plant essential oils against the malarial fever mosquito *Anopheles stephensi*. Trop Biomed..

[33] Rawani A, Banerjee A, Chandra G (2012). Mosquito larvicidal and biting deterrency activity of bud of *Polianthes tuberosa* plants extract against *Anopheles stephensi* and *Culex quinquefasciatus*. J Commun Dis..

[34] Reegan AD, Kinsalin AV, Paulraj MG, Ignacimuthu S (2015). Larvicidal, ovicidal and repellent activities of marine sponge *Cliona celata* (Grant) extracts against *Anopheles stephensi* Liston (Diptera: Culicidae). Asian Pac J Trop Med..

[35] Swathi S, Murugananthan G, Ghosh S, Pradeep A (2012). Larvicidal and repellent activities of ethanolic extract of *Datura stramonium* leaves against mosquitoes. Int J Pharm Phytochem Res..

[36] Das M, Ansari M (2003). Evaluation of repellent action of *Cymbopogan martinii martinii* Stapf var sofia oil against *Anopheles sundaicus* in tribal villages of Car Nicobar Island, Andaman & Nicobar Islands, India. J Vector Borne Dis..

[37] Singh S, Raghavendra K, Dash A (2009). Evaluation of hexane extract of tuber of root of *Cyperus rotundus* Linn (Cyperaceae) for repellency against mosquito vectors. J Parasitol Res..

[38] Kamaraj C, Rahuman AA, Bagavan A, Elango G, Zahir AA, Santhoshkumar T (2011). Larvicidal and repellent activity of medicinal plant extracts from Eastern Ghats of South India against malaria and filariasis vectors. Asian Pac J Trop Med..

[39] Kumar KP, Murugan K, Kovendan K, Kumar AN, Hwang J-S, Barnard DR (2012). Combined effect of seaweed (*Sargassum wightii*) and *Bacillus thuringiensis* var *israelensis* on the coastal mosquito, *Anopheles sundaicus*. Tamil Nadu India Sci Asia..

[40] Madhiyazhagan P, Murugan K, Kumar AN, Nataraj T (2014). Extraction of mosquitocidals from *Ocimum canum* leaves for the control of dengue and malarial vectors. Asian Pac J Trop Med..

[41] Auysawasdi N, Chuntranuluck S, Phasomkusolsil S, Keeratinijakal V (2016). Improving the effectiveness of three essential oils against *Aedes aegypti* (Linn.) and *Anopheles dirus* (Peyton and Harrison). Parasitol Res..

[42] Phasomkusolsil S, Soonwera M (2011). Comparative mosquito repellency of essential oils against *Aedes aegypti* (Linn.), *Anopheles dirus* (Peyton and Harrison) and *Culex quinquefasciatus* (Say). Asian Pac J Trop Biomed..

[43] Trongtokit Y, Rongsriyam Y, Komalamisra N, Apiwathnasorn C (2005). Comparative repellency of 38 essential oils against mosquito bites. Phytother Res..

[44] Tawatsin A, Wratten SD, Scott RR, Thavara U, Techadamrongsin Y (2001). Repellency of volatile oils from plants against three mosquito vectors. J Vector Ecol..

[45] Phasomkusolsil S, Soonwera M (2010). Insect repellent activity of medicinal plant oils against *Aedes aegypti* (Linn.), *Anopheles minimus* (Theobald) and *Culex quinquefasciatus* Say based on protection time and biting rate. Southeast Asian J Trop Med Public Health..

[46] Trongtokit Y, Curtis CF, Rongsriyam Y (2005). Efficacy of repellent products against caged and free flying *Anopheles stephensi* mosquitoes. Southeast Asian J Trop Med Public Health..

[47] Sritabutra D, Soonwera M, Waltanachanobon S, Poungjai S (2011). Evaluation of herbal essential oil as repellents against *Aedes aegypti* (L.) and *Anopheles dirus* Peyton & Harrion. Asian Pac J Trop Biomed..

[48] Tuetun B, Choochote W, Kanjanapothi D, Rattanachanpichai E, Chaithong U, Chaiwong P (2005). Repellent properties of celery, *Apium graveolens* L, compared with commercial repellents, against mosquitoes under laboratory and field conditions. Trop Med Int Health..

[49] Abiy E, Gebre-Michael T, Balkew M, Medhin G (2015). Repellent efficacy of DEET, MyggA, neem (*Azedirachta indica*) oil and chinaberry (*Melia azedarach*) oil against *Anopheles arabiensis*, the principal malaria vector in Ethiopia. Malar J..

[50] Karunamoorthi K, Mulelam A, Wassie F (2008). Laboratory evaluation of traditional insect/mosquito repellent plants against *Anopheles arabiensis*, the predominant malaria vector in Ethiopia. Parasitol Res..

[51] Karunamoorthi K, Ilango K, Murugan K (2010). Laboratory evaluation of traditionally used plant-based insect repellent against the malaria vector *Anopheles arabiensis* Patton (Diptera: Culicidae). Parasitol Res..

[52] Solomon B, Gebre-Mariam T, Asres K (2012). Mosquito repellent actions of the essential oils of *Cymbopogon citratus*, *Cymbopogon nardus* and *Eucalyptus citriodora*: evaluation and formulation studies. J Essent Oil Bearing Plants..

[53] Alwala O, Wanzala W, Inyambukho R, Osundwa E, Ndiege I (2010). Characterization and evaluation of repellent effect of essential oil of *Mangifera indica* L. from Kenya. J Essent Oil Bearing Plants..

[54] Seyoum A, Pålsson K, Kung’a S, Kabiru E, Lwande W, Killeen G (2002). Traditional use of mosquito-repellent plants in western Kenya and their evaluation in semi-field experimental huts against *Anopheles gambiae*: ethnobotanical studies and application by thermal expulsion and direct burning. Trans R Soc Trop Med Hyg..

[55] Mayeku W, Omollo N, Odalo O, Hassanali A (2014). Chemical composition and mosquito repellency of essential oil of *Conyza newii* propagated in different geographical locations of Kenya. Med Vet Entomol..

[56] Birkett MA, Hassanali A, Hoglund S, Pettersson J, Pickett JA (2011). Repellent activity of catmint, *Nepeta cataria*, and iridoid nepetalactone isomers against Afro-tropical mosquitoes, ixodid ticks and red poultry mites. Phytochemistry..

[57] Innocent E, Hassanali A (2014). Constituents of essential oils from three plant species used in traditional medicine and insect control in Tanzania. J Herb Spice Med Plants..

[58] Barnard DR (1999). Repellency of essential oils to mosquitoes (Diptera: Culicidae). J Med Entomol..

[59] Kweka EJ, Mosha F, Lowassa A, Mahande AM, Kitau J, Matowo J (2008). Ethnobotanical study of some of mosquito repellent plants in north-eastern Tanzania. Malar J..

[60] De Paula JP, Gomes-Carneiro MR, Paumgartten FJ (2003). Chemical composition, toxicity and mosquito repellency of *Ocimum selloi* oil. J Ethnopharmacol..

[61] Nour AH, Elhussein SA, Osman NA, Nour AH (2009). Repellent activities of the essential oils of four Sudanese accessions of basil (*Ocimum basilicum* L.) against Anopheles mosquito. J Appl Sci..

[62] Tavassoli M, Shayeghi M, Abai MR, Vatandoost H, Khoobdel M, Salari M (2011). Repellency effects of essential oils of Myrtle (*Myrtus communis*), Marigold (*Calendula officinalis*) compared with DEET against *Anopheles stephensi* on human volunteers. Iranian J Arthropod Borne Dis..

[63] Younoussa L, Nukenine EN, Danga SPY, Esimone CO (2016). Repellent activity of the creams formulated from *Annona senegalensis* and *Boswellia dalzielii* leaf fractions and essential oils against *Anopheles gambiae* (Diptera: Culicidae). Asian Pac J Trop Dis..

[64] Konan Y, Sylla M, Doannio J, Traoré S (2003). Comparison of the effect of two excipients (karite nut butter and vaseline) on the efficacy of *Cocos nucifera*, *Elaeis guineensis* and *Carapa procera* oil-based repellents formulations against mosquitoes biting in Ivory Coast. Parasite..

[65] Freeman BC, Beattie GA (2008). An overview of plant defenses against pathogens and herbivores. Plant Health Instructor..

[66] Morehead JA. Efficacy of organic insecticides and repellents against brown marmorated stink bug in vegetables. https://vtechworks.lib.vt.edu/handle/10919/71810. Accessed 28 Mar 2016.

[67] Yeh R-Y, Shiu Y-L, Shei S-C, Cheng S-C, Huang S-Y, Lin J-C (2009). Evaluation of the antibacterial activity of leaf and twig extracts of stout camphor tree, *Cinnamomum kanehirae*, and the effects on immunity and disease resistance of white shrimp, *Litopenaeus vannamei*. Fish Shellfish Immunol..

[68] Grognet J (1990). Catnip: its uses and effects, past and present. Canadian Vet J..

[69] Abu-Darwish MS, Abu-Dieyeh ZH, Mufeed B, Al-Tawaha ARM, Al-Dalain SYA (2009). Trace element contents and essential oil yields from wild thyme plant (*Thymus serpyllum* L.) grown at different natural variable environments, Jordan. J Food Agric Environ..

[70] Prado JM, Leal PF, Meireles MAA, Eds. Comparison of manufacturing cost of thyme extract obtained by supercritical fluid extraction and steam distillation. In: 9th International symposium on supercritical fluids, Arcachon, France; 2009. P. 19.

[71] Fabbri A, Hormaza J, Polito V (1995). Random amplified polymorphic DNA analysis of olive (*Olea europaea* L.) cultivars. J Amer Soc Hort Sci..

[72] Yasodha R, Sumathi R, Chezhian P, Kavitha S, Ghosh M (2008). Eucalyptus microsatellites mined in silico: survey and evaluation. J Genetic..

[73] Walle M, Walle B, Zerihun L, Makonnen E (2014). Sedative-hypnotic like effect of the essential oil from the leaves of *Myrtus communis* on mice. Am J Biomed Life Sci..

[74] Miele M, Dondero R, Ciarallo G, Mazzei M (2001). Methyleugenol in *Ocimum basilicum* L. Cv genovese gigante. J Agric Food Chem..

[75] Sadia S, Khalid S, Qureshi R, Bajwa AA (2013). *Tagetes minuta* L., a useful underutilized plant of family Asteraceae: a review. Pak J Weed Sci Res..

[76] Bahuguna V (1997). Silviculture and management practices for cultivation of *Azadirachta indica* (Neem). Indian Forester..

[77] Lara M, Gutierrez J, Timon M, Andrés A (2011). Evaluation of two natural extracts (*Rosmarinus officinalis* L. and *Melissa officinalis* L.) as antioxidants in cooked pork patties packed in MAP. Meat Sci..

[78] Singh AK, Dhamanigi SS, Asad M (2009). Anti-stress activity of hydro-alcoholic extract of *Eugenia caryophyllus* buds (clove). Indian J Pharmacol..

[79] Acar Ü, Kesbiç OS, Yılmaz S, Gültepe N, Türker A (2015). Evaluation of the effects of essential oil extracted from sweet orange peel (*Citrus sinensis*) on growth rate of tilapia (*Oreochromis mossambicus*) and possible disease resistance against *Streptococcus iniae*. Aquaculture..

[80] Durrani F, Ismail M, Sultan A, Suhail S, Chand N, Durrani Z (2006). Effect of different levels of feed added turmeric (Curcuma longa) on the performance of broiler chicks. J Agric Biol Sci..

[81] Moore S, Lenglet A, Hill N, Debboun M, Frances SP, Strickman D (2006). Plant-based insect repellents. Insect repellents: principles, methods and uses.

[82] Pappenberger B, Geier M, Boeckh J, Foundation Ciba (1996). Responses of antennal olfactory receptors in the yellow fever mosquito *Aedes aegypti* to human body odours. Olfaction in mosquito-host interactions.

[83] Kwon Y, Kim SH, Ronderos DS, Lee Y, Akitake B, Woodward OM (2010). Drosophila TRPA1 channel is required to avoid the naturally occurring insect repellent citronellal. Curr Biol..

[84] Kongkaew C, Sakunrag I, Chaiyakunapruk N, Tawatsin A (2011). Effectiveness of citronella preparations in preventing mosquito bites: systematic review of controlled laboratory experimental studies. Trop Med Int Health..

[85] Soderlund DM, Bloomquist JR (1989). Neurotoxic actions of pyrethroid insecticides. Annu Rev Entomol..

[86] Ditzen M, Pellegrino M, Vosshall LB (2008). Insect odorant receptors are molecular targets of the insect repellent. DEET Sci..

[87] Tyagi B, Veer V, Gopalakrishnan R (2016). Advances in vector mosquito control technologies, with particular reference to herbal products. Herbal insecticides, repellents and biomedicines: effectiveness and commercialization.

[88] Lupi E, Hatz C, Schlagenhauf P (2013). The efficacy of repellents against Aedes, Anopheles, Culex and Ixodes spp. A literature review. Trav Med Infect Dis..

[89] Yogananth N, Anuradha V, Ali MYS, Muthezhilan R, Chanthuru A, Prabu MM (2015). Chemical properties of essential oil from *Rhizophora mucronata* mangrove leaf against malarial mosquito *Anopheles stephensi* and filarial mosquito *Culex quinquefasciatus*. Asian Pac J Trop Med..

